# A phage-targeting strategy for the design of spatiotemporal drug delivery from grafted matrices

**DOI:** 10.1186/1755-1536-4-7

**Published:** 2011-02-17

**Authors:** Ritsuko Sawada, Carrie Y Peterson, Ana Maria Gonzalez, Bruce M Potenza, Barbara Mueller, Raul Coimbra, Brian P Eliceiri, Andrew Baird

**Affiliations:** 1 Department of Surgery, Division of Trauma, Surgical Critical Care and Burns, University of California San Diego School of Medicine, 200 W. Arbor Dr., San Diego, CA 92103-8236 USA; 2 MabVax Therapeutics, Inc., 11588 Sorrento Valley Rd, Suite 20, San Diego, CA 92121 USA; 3 College of Medical and Dental Sciences, University of Birmingham, Edgbaston, Birmingham, B15 2TT, UK; 4 Torrey Pines Institute for Molecular Studies, 3550 General Atomics Court, San Diego, CA 92121-1122 USA

## Abstract

**Background:**

The natural response to injury is dynamic and normally consists of complex temporal and spatial cellular changes in gene expression, which, when acting in synchrony, result in patent tissue repair and, in some instances, regeneration. However, current therapeutic regiments are static and most rely on matrices, gels and engineered skin tissue. Accordingly, there is a need to design next-generation grafting materials to enable biotherapeutic spatiotemporal targeting from clinically approved matrices. To this end, rather then focus on developing completely new grafting materials, we investigated whether phage display could be deployed onto clinically approved synthetic grafts to identify peptide motifs capable of linking pharmaceutical drugs with differential affinities and eventually, control drug delivery from matrices over both space and time.

**Methods:**

To test this hypothesis, we biopanned combinatorial peptide libraries onto different formulations of a wound-healing matrix (Integra^®^) and eluted the bound peptides with 1) high salt, 2) collagen and glycosaminoglycan or 3) low pH. After three to six rounds of biopanning, phage recovery and phage amplification of the bound particles, any phage that had acquired a capacity to bind the matrix was sequenced.

**Results:**

In this first report, we identify distinct classes of matrix-binding peptides which elute differently from the screened matrix and demonstrate that they can be applied in a spatially relevant manner.

**Conclusions:**

We suggest that further applications of these combinatorial techniques to wound-healing matrices may offer a new way to improve the performance of clinically approved matrices so as to introduce temporal and spatial control over drug delivery.

## Background

Synthetic grafts for full-thickness acute and chronic wounds have emerged as an important tool in the surgical management of cutaneous injuries [1-5]. Recognizing that significant improvements are still needed, several investigators have proposed that a combination of matrices with biotherapeutics would be an important direction in the development of next generation grafts [6-11]. Although it may be possible to create new matrices *de novo*, one promising approach has involved adding biotherapeutics and gene-based medicines to commonly used and clinically approved matrices before their deployment [1,2,6,7,10-16]. Athough this approach builds on a significant amount of clinical experience, the results have unfortunately been variable, and furthermore, the approach is not amenable to treatment after graft placement. For example, gene-activated matrices (GAM) [17-21] require preformulation of genes within collagen matrices and, although we have used wound-healing matrices (Integra^®^; Integra LifeSciences, Plainsboro, NJ, USA) in animal models of wound healing to evaluate exogenous gene delivery at the site of injury after deployment [21,22], the response with different strategies has been variable, presumably because of changes in DNA/DNA vector-fixation capacity in already deployed matrices.

A crucial factor in the inclusion of candidate biotherapeutics into the wound bed is the assumption that factors, in particular growth factors, will have the necessary bioavailability to elicit a biologic response at the right place and at the right time [6,9,12]. Accordingly, the disappointing performance of recombinant growth factors and gene medicines in various clinical paradigms [23-30] may simply reflect the pharmacokinetics of these factors in the wound bed, their stability in a protease-rich environment, and their spatial distribution on matrices. Although the use of synthetic and semisynthetic grafts may have achieved widespread clinical use, there remains an important challenge to transform these first-generation matrices into dynamic, spatiotemporally sensitive, next-generation drug-deployment vehicles.

One way to monitor the changes in spatiotemporal responsiveness to therapeutics has involved analyzing the wound-bed genome [31-34], transcriptome [35-38] and proteome [35,39] for clues as to what genes are active, and when and where. Another approach involves evaluating changes in endogenous gene expression non-invasively and in real time [40-42]. To this end, we have shown that it is possible to monitor non-invasively any changes in vascularization and permeability after grafting [22], and that both endogenous and exogenous gene expression can be monitored noninvasively after placement of tissue grafts [21]. Because the identification of when and where drug targets are expressed defines the parameters of spatiotemporal delivery, we sought to describe two endpoints, angiogenesis and inflammation, to guide our drug-delivery efforts.

In the present study, we tested whether combinatorial tools such as phage display [43] could be used to help design next-generation matrices capable of drug delivery. If so, we reasoned that a genetic selection of active peptides might help identify unique classes of peptides with different affinities and specificities, whose activities are dependent, *a priori*, on the specific protocol used for their selection. We first focused on characterizing the dynamic nature of the endogenous gene expression in the wound bed in an attempt to characterize aspects of the temporal and spatial kinetics of tissue responsiveness. We then focused on identifying specific peptide motifs that could be deployed to target the graft with specific, and presumably differential, affinities. In designing the targeting strategy for these proof-of-concept experiments, we selected a clinically approved graft that is 1) in wide use for both full-thickness burns and chronic wounds, 2) a biosynthetic formulation that is stable at room temperature, 3) suitable for combinatorial screening and 4) amenable to biopanning using phage display. We show how an Integra^®^-based matrix that is a composite of modified type I bovine collagen and chondroitin sulfate glycosaminoglycan (CS-GAG), can be used to select for peptide targeting. Moreover, having previously used this matrix to define the kinetics of vascular permeability and neovascularization in a full-thickness wound, we show that there are temporal and spatial changes in gene expression that accompany tissue repair, which specifically need targeting at different times and different locations. These data provide the first systematic evaluation of phage display to identify peptide motifs for biotherapeutic targeting to the wound bed. We also describe the screening protocols used to detect, identify and validate these peptides. The possibility that these findings could serve as a foundation to evolve static synthetic matrices into dynamic grafts that provide temporal and spatial control over drug delivery is discussed.

## Results

**Figure 1:**
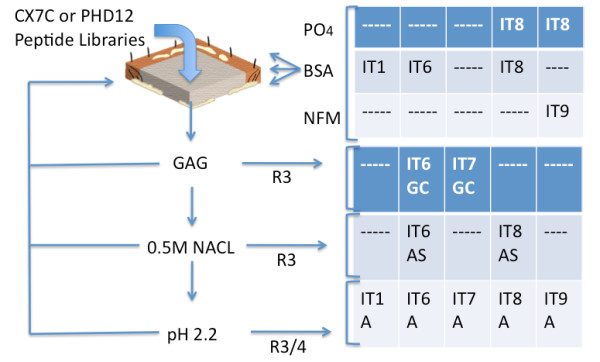
**Summary of phage screening strategy**. Libraries of targeted phage were added to either intact matrix (IT1), sonicated immobilized matrix (IT6, IT7 and IT9) or thin sections of matrix (IT8). The blocking strategies with phosphate buffer (P04), bovine serum albumin (BSA) or non fat milk (NFM) were deployed as indicated, and eluates from samples treated with chondroitin sulfate, high salt or high acid, which were processed over three to four rounds (R3, R3/4) of selection before sequence analyses, were used.

We evaluated and subsequently deployed several strategies to identify peptides capable of interacting with the Integra^® ^matrices. These included the use of 1) two different peptide libraries as starting materials including the PHD-C7C and PHD12 libraries; 2) different formulations of the target including intact, solubilized or cryostat-sectioned matrices for biopanning; 3) different elution buffers including chondroitin sulfate, low pH and high salt; and lastly 4) different blocking buffers including bovine serum albumin (BSA), phosphate-buffered saline containing 0.1% Tween (PBS-T), and non-fat milk (NFM) to limit non-specific binding of phage These variations would allow us to select particles that display different classes of peptides with different affinities for the target matrix. The approaches used are detailed in Table 1 and summarized schematically in Figure 1.

**Table 1:** Summarized biopanning strategies used^a ^for the identification of Integra^® ^(IT) matrix targeting peptides from New England Biolabs libraries

Protocol	IT1BA	IT6BA	IT6BAS	IT6BGC	IT7BA	IT7BCG	IT8BA	IT8BAS	IT8PA	IT8PAS	IT9MA	IT9PA
Target	Matrix	Sonicated	Sonicated	Sonicated	Sonicated	Sonicated	Sections	Sections	Sections	Sections	Sonicated	Sonicated
Library	C7C	C7C	C7C	C7C	PHD12	PHD12	C7C	C7C	C7C	C7C	PHD12	PHD12
Block	BSA	BSA	BSA	BSA	BSA	BSA	BSA	BSA	PO_4_	PO_4_	NFM	PO_4_
Elution	Acid	Acid	Acid/salt	GAG	Acid	GAG	Acid	Acid/salt	Acid	Acid/salt	Acid	Acid
Selection rounds	3 to 5	3	3	3	3	3	4	3	4	3	3	3
Colonies analyzed	50	15	15	15	15	15	30	30	30	30	30	30
Peptide ID	Table 2	Table 2	Table 2	Table 2	Table 2	Table 2	Table 3	Table 3	Table 3	Table 3	Table 3	Table 3
Phylogeny	Figure 3	Figure 4A	Figure 4A	Figure 5A	Figure 4B	Figure 5B	Figure 6A,C	Figure 6A	Figure 6B,D	Figure 6B	Figure 7	Figure 7

^a^Four distinct strategies were evaluated (IT1, IT7, IT8 and IT9), which involved changing the blocking buffer (phosphate-buffered saline with Tween, bovine serum albumin (BSA) and non-fat milk (NFM)), the elution conditions (acid, acid/salt and glaycosaminoglycan), the target (matrix, sonicated samples and thin sections) and the number of rounds (three or four) of biopanning.

### Temporal and spatial assessment of vascular remodeling and inflammation in vascular endothelial growth factor receptor-luciferase and Smad 2/3 binding element-luciferae transgenic mice

**Figure 2:**
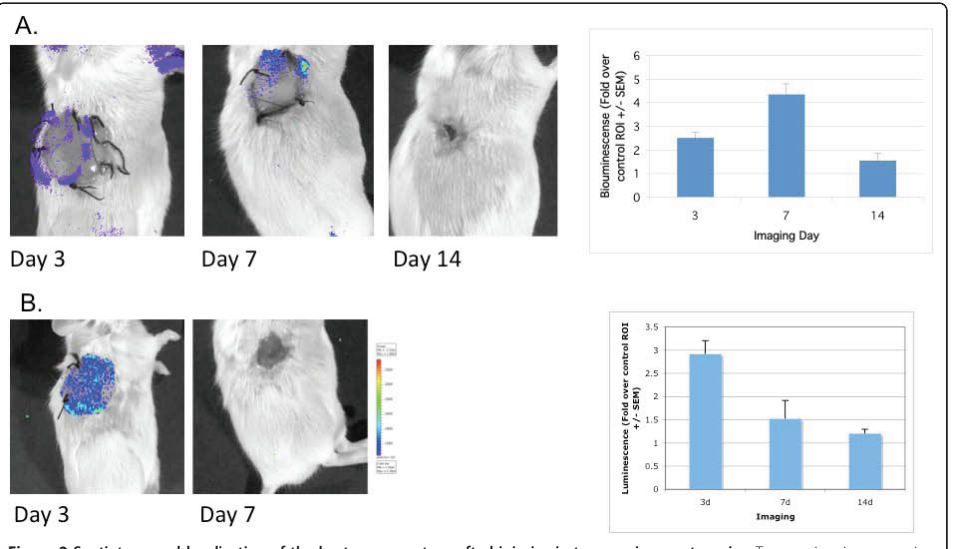
**Spatiotemporal localization of the host response to grafted injuries in transgenic reporter mice**. Transgenic mice expressing the firefly luciferase reporter under the regulation of either **(A) **the vascular endothelial growth factor receptor 2 promoter or **(B) **the Smad binding element promote were analyzed for the kinetics of the vascular remodeling and scarring response, respectively, 3, 7 and 14 days after placement of a full-thickness Integra^® ^graft. Transgenic expression of luciferase was imaged and bioluminescence quantified by selecting a circular region of interest (ROI) at the grafting site and comparing it to an ROI at a site remote form the injury.

To assess spatiotemporal control of angiogenesis, we examined the activation of the vascular endothelial growth factor receptor (VEGFR)2 gene (*vegfr*2) in a transgenic mouse that expresses firefly luciferase under the regulation of the VEGFR2 promoter. Whereas Zhang *et al. *[44] used this model to assess contact sensitivity, we sought to define the kinetics of VEGF-dependent responsiveness in a full-thickness wound grafted with Integra^® ^to determine its correlation with the vascular remodeling seen in our injury model [21,22]. Representative images of grafted wounds in each of these mice localized VEGFR2-controlled luciferase gene expression to the rim of the wound, with a peak of gene expression at 7 days (Figure 2A). As expected from our previous immunohistochemical studies [21,22], the localization of the *vegfr2 *gene expression in the angiogenic rim corresponded to the region of most active angiogenesis in these grafts. Over the 14-day period in which these full-thickness wounds consolidated, contracted and healed, peak VEGFR2 promoter activation was recorded on day 7 (Figure 2A). These observations suggest that the VEGFR2 promoter activation of luciferase in intact transgenic mice can be used as a model to both localize the distribution of vascular remodeling events and quantify the kinetics of expression.

Signaling through the transforming growth factor (TFF)-β and Smad 2/3 pathway, in contrast, has been associated in various *in vitro *and *in vivo *models with inflammation and scarring [45,46]. The binding element for the Smad 2 and 3 transcription factors can be used to monitor *smad2/3 *gene activation. Therefore, to determine the kinetics of activation of this pathway, we examined the activation of this Smad Binding Element (SBE) promoter in a luciferase transgenic mouse after a full-thickness wound and graft. Results showed luciferase activity at 3 days distributed across the wound bed, which resolved by day 7 (Figure 2B). Quantification of the luciferase activity showed a peak as early as post-operative day 3 (the earliest day examined), which declined through the 14-day time course. Whereas TGF-β-Smad2/3 pathway signaling is generally associated with scar formation, the determinants for which are thought to be a late response of the wound bed, these data suggest that SBE signaling may be an early response.

### Screening strategy for identification of matrix targeting peptides

In light of the spatial and temporal changes seen in the expression of endogenous genes after injury, we sought to design a drug-immobilization strategy that would enable biotherapeutic absorption at specific sites within target matrices. It is our long-term goal to modulate biotherapeutic affinities to matrices so as to introduce intrinsic temporal and spatial control of biotherapeutic release. With this end in mind, we designed and deployed a multifaceted phage display screening strategy to identify peptide motifs capable of differential binding to Integra^® ^matrices. By comparing the results obtained from PHD-C7C and PHD12 phage libraries, we hoped to generate different classes of linker candidates for biotherapeutic delivery. By evaluating specific blocking and elution strategies, we reasoned that we could preferentially select peptides with differential affinities and hence increase the heterogeneity of the genetic selection of IT targeting peptide candidates. This overall strategy is described in Figure 1 and summarized in Table 1, and the identity of peptides recovered is presented in Table 2 and Table 3.

**Table 2:** Identification of Integra^® ^(IT) matrix targeting peptides from libraries eluted by acid, acid salt and chondroitin sulfate^a^

**IT1**^b^	R3 (pH 2)	**IT6**^c^	R3 (pH 2)	IT6	**R (GAG**^d^**)**	**IT7**^c^	R3 (pH 2)	IT7	R3 (GAG)
BSA	IT1 (Figure 3)	BSA^e^	IT6-BA (Figure 4A)	BSA	IT6-CG (Figure 4B)	BSA	IT7-BA (Figure 5A)	BSA	IT7-CG (Figure 5B)
3.2	CTE**S**APYFC	A-1	CNPLHRQHC	CG-1	CLSTSSKSC	BA-1	HWHDWMWSWRRD	CG-1	AYYPQNHKSNAE
3.4	CPD**A**NNGNC	A-2	CFKHSSHQC	CG-2	CLSTSSKSC	BA-2	SMWPWYYSQWAR	CG-2	APQYQHNQATHT
3.7	CNM**A**Q**T**NMC	A-3	CPPTPLSLC	CG-3	CLSTSSKSC	BA-3	TLGDRYSTKHPI	CG-3	SITWTHHPGALQ
3.8	CPN**A**NLGTC	A-4	CINASKPLC	CG-4	CQTSANTQC	BA-4	SFSTMNTAPGGS	CG-4	AGLHPRSLESLP
4.2	CIN**S**FYAQC	A-5	CNRMVQPMC	CG-5	CGVPAGSTC	BA-5	WYMPWWSAGQAA	CG-5	HPGNRSLDPLNH
4.3	CKS**A**I**S**SSC	A-6	CNLALTQAC	CG-6	CLSTSSKSC	BA-7	QKKIRKRPHVKR	CG-6	LLADTTHHRPWT
4.4	CVPQY**S**SQC	A-7	CFKHSSHQC	CG-7	CLSTSSKSC	BA-8	GAFHKHHHARLI	CG-7	ATGKPTRLESHV
4.5	CQPKAVNHC	A-8	CQEPRSNAC	CG-8	CLATKLHNC	BA-9	WYMPWWSAGQAA	CG-8	NPSNLYRQPAMT
4.7	CPV**S**P**S**GAC	A-9	CPSHHLESC	CG-9	CLSTSSKSC	BA-10	WNRSPLPDYGAA	CG-9	SKAHDISQRQPP
4.8	CSN**A**SRPFC	A-10	CNPLHRQHC	CG-10	CDGVSTKHC	BA-11	SLWQRWFPVLDH	CG-10	VNRIPGENLSSP
4.10	CNP**A**L**S**THC	A-11	CSKTFPVRC	CG-11	CLSTSSKSC	BA-12	HWHDWMWSWRRD	CG-11	AYYPQNHKSNAE
4.13	CGK**A**GLPLC	A-12	CFKHSSHQC	CG-12	CLSTSSKSC	BA-13	DLALRNPTPSDP	CG-12	SNQPAPALFHQL
4.14	CP**THP**PFQC	-	-	CG-13	CIKNPTKYC	BA-14	SHALPLTWSTAA	CG-13	YSPASKSPVPSL
4.15	CE**S**SAIRYC	AS-1	CFKHSSHQC	CG-14	CLSTSSKSC	BA-15	WHYNSWYRWPVM	CG-14	SVSVGMKPSPRP
4.18	CNNGT**S**RLC	AS-2	CFKHSSHQC	CG-15	CMPSPSLK	-	-	CG-15	SVSVGMKPSPRP
4.19	CP**S**Q**THP**TC	AS-3	CTYPFHASC	-	-	-	-	-	-
4.20	CTNQQRHTC	-	-	-	-	-	-	-	-

^a^The sequences of peptides isolated using the biopanning strategies IT1, IT6 and IT7 are presented along with their code numbers for reference.

^b^IT1 was screened against intact matrix and only acid eluates were evaluated.

^c^IT6 and IT7 used both acid and GAG elutions.

^d^GAG = glycosaminoglycan.

^e^BSA = bovine serum albumin.

**Table 3:** Identification of Integra^® ^(IT) matrix targeting peptides from peptide libraries after bovine serum albumin (BSA), phosphate-buffered saline (PBS) and non-fat milk (NFM) blocking^a^

IT8^b^	R3 (pH 2)	IT8	R3 (NaCl)	IT8	R3 (pH 2)	IT8	R3 (NaCl)	IT8	R4 (pH 2)	IT8	R4 (pH 2)	IT9^c^	R4 (pH 2)
BSA	IT8-BA	BSA	IT8-BAS	PO4	IT8-PA	PO_4_	IT8-PAS-	BSA	IT8-BA	PO_4_	IT8-PA-	NFM	IT9-MA
Figure 6A				Figure 6B				Figure 6C		Figure 6D		Figure 7	
BA1	CVPSSARIC	BAS1	CHMSPRHQS	PA1	CVQSSTQHC	PAS4	CHVTAQRAC	R4BA1	CKGPVSRHC	R4PA1	CGKHDDTYC	MA1	HETFPSPRANSV
BA2	CRPHDSKAC	BAS2	CLPNKQWSC	PA2	CSGHHSLRC	PAS5	CATPEWPPC	R4BA2	CTTSSEHVC	R4PA2	CPTKDLRYC	MA2	HETFPSPRANSV
BA3	CHPEPRSQC	BAS3	CKQPLNNTC	PA3	CPTSQQKVC	PAS6	CPNLMNTRC	R4BA3	CSTTMKTSC	R4PA3	CTSSGNRYC	MA3	HETFPSPRANSV
BA4	CVEKRPRQC	BAS4	CTVTPRHLC	PA4	CNSTHPRAC	PAS8	CTKSSPPRC	R4BA4	CDNKRSPAC	R4PA4	CTIKTNLQC	MA4	HETFPSPRANSV
BA5	CFMDYRNLC	BAS5	CDNTSKTQC	PA5	CNRLESHLC	PAS9	CTQTTVASC	R4BA5	CKLNYPNAC	R4PA5	CHSTAKSAC	MA5	SQIDYATGPRQA
BA6	CSHSVQPFC	BAS6	CASTTAACC	PA6	CTNPHRSQC	PAS10	CDQSKTIAC	R4BA7	CQFSKSQSC	R4PA6	CPASKGDFC	MA6	WDTEKASPLSPL
BA7	CQTHNPRQC	BAS7	CLHMDKKRC	PA7	CTKTPWPGC	PAS11	CSRGSMGIC	R4BA8	CTLDTRRDC	R4PA8	CSHRVPHDC	MA7	HETFPSPRANSV
BA8	CDGAPAPLC	BAS8	CMKTPMRSC	PA8	CNRLQGEHC	PAS12	CSPIRGSMC	R4BA10	CPFSSSPSC	R4AP9	CHATPYPKC	MA8	HETFPSPRANSV
BA10	CKTDLQKQC	BAS10	CYKHVGQRC	PA9	CPTPTGRYC	PAS13	CSHTGHHQC	R4BA11	CPSMSHHQC	R4PA10	CDSSRHTHC	MA10	DHTGKSPGLFHN
BA11	CGPFPQPHC	BAS11	CHLSPFKSC	PA10	CVPTAMSNC	PAS14	CHEPTTMAC	R4BA12	CSASTQSFC	R4PA11	CSRLSQEYC	-	-
BA12	CSFHGPGPC	BAS12	CTTSKYRDC	PA11	CSLARPNEC	PAS15	CSRADLTTC	R4BA13	CPLKGLATC	R4PA12	CTGKQYPQC	-	-
BA13	CSTNQTPTC	BAS13	CTATGLSNC	PA12	CVRTPFSMC	-	-	R4BA14	CTGKPLKTC	R4PA13	CGMNAFRAC	A1	AHKHKHPGHITA

BA14	CSTSPONSC	BAS14	CPSSMPSRC	PA13	CNNTTPPSC	-	-	R4BA17	CIHMTGYHC	R4PA14	CGMNAFRAC	-	-
BA15	CKLIHNNSC	BAS15	CPATSHTHC	PA15	CTSQQKANC	-	-	R4BA21	CEMTETKHC	R4PA16	CTTKYSTTC	-	-
-	-	-	-	-	-	-	-	R4BA26	CKENWPLIC	R4PA18	CSSDKALVC	-	-
-	-	-	-	-	-	-	-	R4BA30	CSNSPTTMC	R4PA24	CSPRSHLSC	-	-
-	-	-	-	-	-	-	-	-	-	R4PA25	CPPSPMPYC	-	-

^a^The sequences of peptides pulled from the biopanning strategies IT8 and IT9 are presented along with their code numbers for reference.

^b^IT8 was screened against matrix sonicated samples blocked with BSA or PBS-T, and the peptide sequences were generated from both acid and GAG elutions.

^c^IT9 was biopanned from thin sections of matrix blocked with NFM or PBS-T, and only the acid/high salt eluates evaluated.

### Identification of matrix binding peptides eluted under different conditions

The first series of experiments involved screening the PHD-C7C library on intact Integra^® ^bilayer matrix wound dressings. As expected, the recovery of binding particles during the four rounds of biopanning initially decreased, but then subsequently increased 40-fold. We identified two distinct motifs of peptides with this selection strategy: XAXSX and XTHPX (Table 2). The former (XXAXSXX) was detected in sequences recovered from the third round of screening, and the latter (XXTHPXX) was detected in the fourth round.

**Figure 3:**
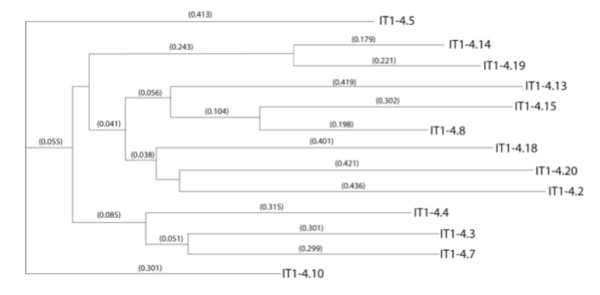
**Phylogenic tree of peptide targeting candidates biopanned from IT1 (PHD-C7C) library screening on intact matrices**. Peptide sequences were obtained after the third and fourth rounds of screening, and analyzed for structural relatedness, using MacVector sequence analysis software. Recovery of particles increased 40-fold over four rounds.

We performed a phylogenic analysis of these Integra^®^-targeting (IT) peptides, which identified one major subfamily represented by peptides IT1-4.8 with two distal sequences (IT1, peptide 4.5 and IT1, peptide 4.20) (Figure 3). Further selection in a fifth round failed to enrich and found increased non-specific adsorption to BSA blocking agents and further screening was discontinued (data not shown).

**Figure 4:**
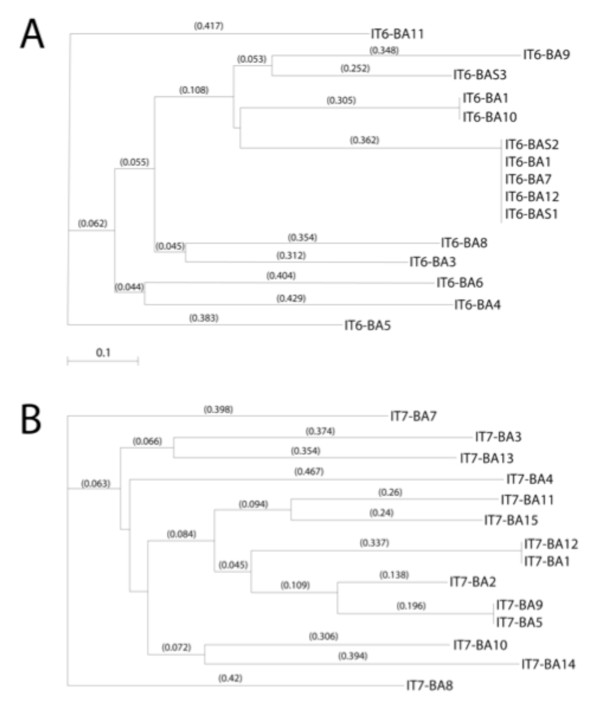
**Phylogenic tree of peptide targeting candidates using acid elution of partially solubilized matrix sonicated samples**. Peptide sequences were obtained after the third round of screening using **(A) **the IT6 protocol, which biopanned using the PHD-C7C library or **(B) **the IT7 protocol, which used the PHD12 library as starting material. In both instances, the phage were recovered from the acid eluates of washed sonicated samples. Sequences were analyzed for structural relatedness using MacVector sequence analysis software. Library collapse was reflected by an increased recovery of 300-fold in IT7 over the three rounds of biopanning.

In IT6 and IT7 screens (Table 2), phages were eluted with either low pH or by displacement with exogenous chondroitin sulfate to obtain particles with different binding capacities. The target for the biopanning was a homogenized and sonicated Integra^® ^matrix that was absorbed to ELISA plates (see Methods). This approach was designed to minimize non-specific sequestration of particles in the highly fenestrated matrix during the biopanning procedure. In one biopanning strategy (IT6), we evaluated peptides recovered from a screen of the PHD-C7C library described above. In a second biopanning set, we analyzed the PHD12 library of linear peptides 12 amino acids long (IT7). In both instances, we were able to recover peptides with distinct motifs, which upon analyses collapsed into separate families (Figure 4A). For example, sequence collapse within the IT6 acid eluate identified one main family of related peptides represented by the sequence of IT6-A2 and a putative consensus sequence of XKHSXH, whereas there were two outlying peptides represented by IT6-A5 and IT6-A11. Peptides eluted with acid/high salt fell into the prime family of the ITA2 peptides recovered, suggesting that the salt wash did not provide any selective advantage. Mining the PHD12 library under these same conditions showed more heterogeneity (Figure 4B) but the sequences nevertheless collapsed to a prime sequence of WMWS represented by the IT7-A1.

**Figure 5:**
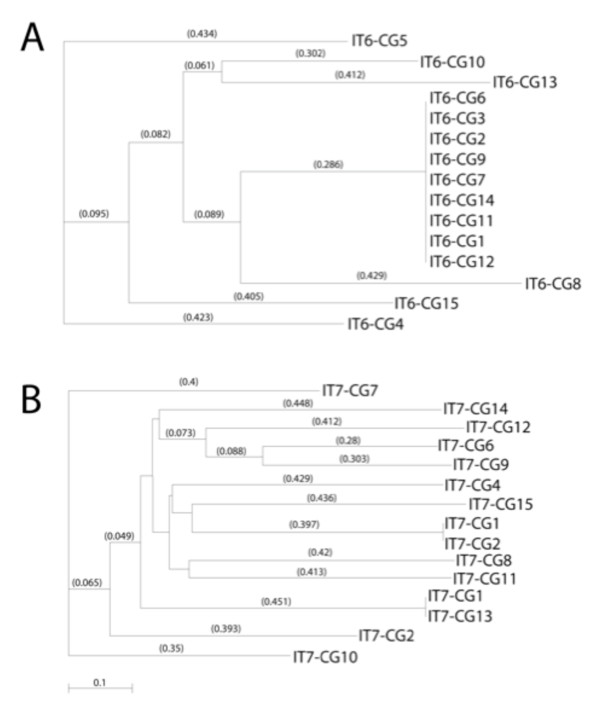
**Phylogenic tree of peptide targeting candidates using chondroitin sulfate elution from matrix sonicated samples**. Peptide sequences were obtained after the third round of screening using **(A) **the IT6 protocol, which biopanned using the C7C library or **(B) **the IT7 protocol, which used the PHD12 library as starting material. In both instances the phage were recovered from the chondroitin sulfate (CS)-glycosaminoglycan (GAG) eluates of washed sonicated matrix samples, which contained CG to remove bound peptides. Sequences were analyzed for structural relatedness using MacVector sequence analysis software. Library collapse was indicated by an increased recovery of 400-fold in IT7 over the three rounds of biopanning.

The collapse of selected peptides from the library is perhaps best illustrated by the sequences obtained from the chondroitin sulfate elutions in IT6. Of 15 sequences, nine were identical and closely related (0.429) to others (IT6-CG8) (Table 2; Figure 5A). The formatted alignments show the importance of Ser/Thr in the selection of these peptides. This was less obvious in the collapse of the PHD12 library in the third round of selection by chondroitin sulfate elution (Figure 5B). Whereas there was one main family of related peptides represented by IT7-3CG, the motif seemed to revolve around the positive charge of histidine (XTHHX) rather then the presence of a hydroxyl group of serine or threonine, yet there are similar alignments in XXLESXX, such as IT7-CG6.

### Identification of matrix-targeting peptides under different blocking conditions

**Figure 6:**
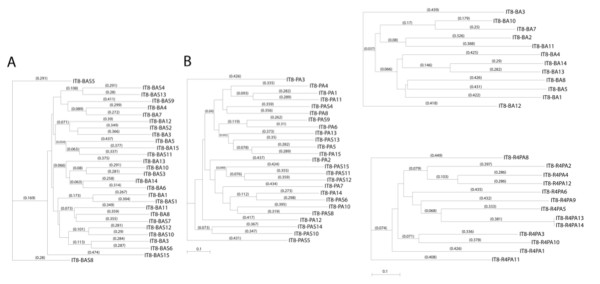
**Phylogenic tree of peptide targeting candidates eluted after bsa or no blocking of matrix sections**. Peptide sequences were obtained after **(A,B) **the third and **(C,D) **fourth (rounds of screening using the IT8 (PHD-C7C Library) biopanning protocol that used a low pH elution (pH2.2) of 10 μm matrix sections that were either (A,C) blocked or (B,D) not with bovine serum albumin. Phage were isolated from single colonies, and the sequences of the peptide inserts (Table 3) were analyzed for structural relatedness using MacVector sequence analysis software.

There was concern that if the IT6 and IT7 protocols above were adapted to evaluate the yields from PHD-C7C and PHD12 libraries, respectively, on sonicated matrix homogenates, selection bias in the combinatorial screens could be generated by the blocking buffer and not just by virtue of sequestration within the matrix, as suspected with IT1 peptides. To address this possibility, we evaluated the identity of binding peptides selected onto thin (10 μm) sections of matrix. Reasoning that thin sections would minimize sequestration within the matrix fenestrations, crysostat = processed matrices were used for biopanning. The results from the four approaches are presented in Table 3. Both the acid and acid/salt eluates of PHD-C7C biopanning in the presence of bovine serum albumin (BSA) identified 14 peptides, which by phylogenic analyses were related, except for two sequences (Figure 6A). Within this family of sequences there were two subfamilies with the consensus sequences XSTSXQX and SXSXH, represented by peptides IT8-3A4 and IT8-3A2, respectively. There was less homology when the biopanning was performed in the absence of BSA (Table 3) and there were three families identified, of which one further segregated into acid and acid/salt motifs (Figure 6B). With a fourth round of screening, the peptides eluting with acid from BSA-blocked sections (Table 3) collapsed further to reveal the XXSXSXSX motif found in peptide IT84A10, whereas the non-blocking fourth-round sequences collapsed towards a similar SSXXTY sequence illustrated by IT8-4B3 (Figure 6C).

**Figure 7:**
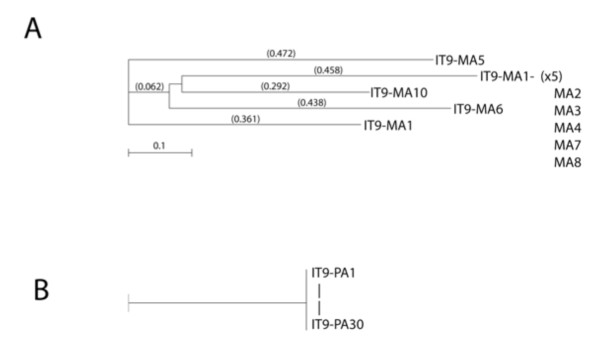
**Phylogenic tree of peptide targeting candidates eluted after non-fat milk (NFM) or no blocking of matrix sonicated samples**. Peptide sequences were obtained after the third round of screening using the IT9 (PHD 12) protocol, with NFM used to block the non-specific binding to sonicated matrix. All 30 clones analyzed in the NFM-free assay corresponded to a single peptide of 12 amino acids: AHKHKPGHITA. Structural relatedness using MacVector sequence analysis software. Recovery of increased 800-fold in IT9 biopanning.

To further address possible biopanning bias that might be introduced by BSA blocking, we compared the screening selection with and without NFM when biopanning against the PHD12 library. After three rounds of processing in IT9, libraries collapsed to the extent that seven of 10 of sequences corresponded to one peptide (HETFPSPRANSV) in the NFM-blocked samples, which also had the XSXXXSX consensus (Figure 7A,B). All 20 sequences obtained from the non-blocked screen corresponded to a sequence not found in previous screens (AHKHKHPGHITA) (Table 3). Phylogenic analyses of the peptides showed that the linkages between the peptides identified could be divided into three classes that, as in other cases, were represented by a central core group of related peptides and two distally related sequences. Their distal sequences corresponded to outliers of the NFM-blocking and non-blocking screens, respectively.

### Evaluation of candidate peptides for binding capacity

**Figure 8:**
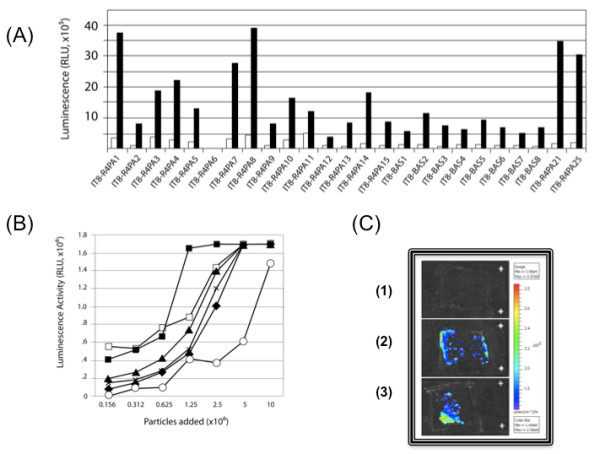
**Peptide screening, dose-response analyses and spatial immobilization of targeted particles onto intact matrices**. Using the methods described in the text, individual phage were tested for either their ability to bind to immobilized components (for example, chondroitin sulfate, collagen 4, bovine serum albumin or to sonicated matrix samples, as illustrated by **(A) **binding of IT8 peptides to immobilized chondroitin sulfate or **(B) **dose-response analyses to specific peptides binding to sonicated matrix samples immobilized in ELISA wells. Spatial binding to matrix sections was established by placing targeted particles as **(C) **continuous lines along two sides (sample 2) or as a **dot **(sample 3). Phosphate-buffered saline alone was negative (sample 1). In all instances, binding of particles was assessed by chemiluminescence.

The strategy to confer temporal and spatial control to drug release on the matrices involves the use of peptides with differential affinities to components of the deployed graft. To this end, we evaluated peptide binding to the matrix relative to controls by direct incubation onto the target. Representative results for different targeting peptides are presented in Figure 8. The initial screen involved direct binding analyses of the targeted particles as illustrated for the IT8 peptides recovered in the presence or absence of blocking BSA (Figure 8A). Under the ELISA conditions used (see Methods), background binding was low and there was an approximately 10^5 ^increase in binding. Although all of the peptides selected as candidates showed specific binding, some (for example, IT8-R4PA21) seemed to have greater capacity. This affinity is evaluated in dose response curves which, showed a dose -dependent increase in binding that reflects the affinity the peptides confer to the particles (Figure 8B). In this specific example, binding of peptides selected by chondroitin sulfate displacement was evaluated in an ELISA using the glycosaminoglycan to capture targeted phage. Although there was a 10^5^-fold increase in saturable binding, the affinities were narrow and detectable over a two- to three-fold change in concentrations. In-depth analysis of specificity, capacity, affinity and avidity of all the peptide candidates is underway on matrix, sonicated samples and GAG-coated plates to select targeting leads for evaluation in preclinical studies.

### Validation of peptides on intact matrix

Although changes in affinity might meet the long-term goal to confer temporal control over delivery, we also wanted to establish whether the targeting peptides could be compatible with spatial control. To this end, we sought to decorate matrices so that they could be designed for drug delivery in a spatially relevant manner, for example in the angiogenic ring described in Figure 2A. Targeted phage can be placed onto sections so as to absorb to specific loci (Figure 8C). In the case of targeting VEGFR2, particles were placed in the periphery of the matrix. In one example (Figure 8C, number 2) the peptide IT6-A7, derived form the PHD-C7C library and with the sequence CFKHSSHQC displayed on the phage, was placed as parallel lines on sections, and the lines were clearly visualized by immunohistochemistry. In another example (Figure 8C, number3), we demonstrated focal immobilization and hence, potential for targeting specific loci with the IT6-A7 particle, identified from the PHD12 library, with the targeting sequence SQIDYATGPRQA displayed on the phage. When placed as points on sections, the position (lower left corner) of the IT6-A7 particle was clearly visualized by immunohistochemistry. This spatial immobilization, coupled with the temporal differences in binding conferred by differential affinities, provides the first step towards the design of spatially and temporally controlled drug delivery from matrices.

## Discussion

In this paper, we provide data supporting the use of phage display to identify targeting peptides for Integra^®^, a surgical dressing used for the treatment of full-thickness burns and wounds and approved by the US Food and Drug Administration. We evaluated several conditions for phage targeting of the grafting material including 1) different starting peptide libraries, 2) different elution strategies, 3) different blocking strategies and 3) different formulations of the matrix material to facilitate selection of peptides with different binding characteristics. Although our study focused on the use of Integra^® ^as a matrix product, any matrix formulation could be subjected to similar evaluation and explored for possible targeting peptides. In fact, it is possible that alternative proteins to CS-GAG may have peptide sequences with widely different affinities. To this end, it is important to note that the peptides identified here represent first-generation sequences that can be optimized and converted to leads by further exploration of their affinities, specificities and relative elution characteristics.

We demonstrate an application of phage display towards mining the range of various binding sites presumably present in a collagen-glycosaminoglycan matrix such as Integra^®^. Further investigation is needed to determine whether these binding motifs can be used for the design of modified biotherapeutics to complement the biologic activity of cutaneous wound healing and tissue repair. Thus, although the biologic significance of these binding sites remains to be determined *in vivo*, the identification of these candidate motifs is a necessary first step towards the design of spatiotemporal matrix-deployed biotherapeutics.

Integra^® ^is an engineered bilayer matrix that consists primarily of GAG with denatured bovine collagen. This acellular synthetic dressing has clinical applications in full-thickness burn injury and wounds, and more limited uses in tissue regeneration. The capacity to combine the use of such a wound dressing with a molecular technique such as phage display presents the possibility of designing strategies that can exploit the different pharmacokinetic properties of the peptides identified. For example, we anticipate that a slow and stable release of growth factors, cytokines and other remodeling factors could be used to modulate the natural course of tissue repair. The matrix would act to stabilize the delivery of the pharmaceutical at the site of the wound bed in the precise location at which its activity can most influence wound healing (that is, angiogenic factors on the periphery and superficial surfaces of the matrix, and scar-limiting factors throughout). When key events in wound repair occur, such as granulation tissue growth and protease release into the matrix, local conditions would allow the release of the pharmaceutical drug into the wound to facilitate healing. Additionally, pharmaceuticals could be designed that would be released when exposed to wound exudates, leading to the protease-mediated degradation of substances that impede wound healing. Alternatively, the targeting peptides could be used to target nanoparticles, liposomes and viruses to matrices for gene delivery to the wound bed.

Phage display is a widely used combinatorial technique that has been used extensively since its discovery 25 years ago by Smith [47]. Numerous groups have used it to identify interacting peptides and their binding partners in simple, complex and even *in vivo *environments [29,43,48-59], but to our knowledge, it has never been deployed for graft targeting *per se*, although a number of integrin targeting peptides have been identified and exploited [51,54,60,61]. Phage display has also been used to identify binding peptides for stem cells, immune cells, bacteria and recombinant proteins [43], and of particular interest to the studies described here, clotted plasma [57]. In this latter study, clotted plasma, an essential element of wound closure, was subjected to phage-library screening to identify peptide motifs that localized to the injured tissue and fibrin/fibronectin-containing tumors. Although our studies focused more on the experimental conditions for peptide identification than the specific binding motifs for a biosynthetic matrix, it is clear that the integrity of these motifs within a wound bed are changing and undergoing active remodeling, and may be altered. Accordingly, phage display may also be used to identify changes in binding sites as they change in the wound bed, by combining the approach described here with protocols involving *in vivo *biopanning paradigms, used by other laboratories [48,58,59]. This work is currently underway in our laboratories.

Although we began with the goal of using the discovered peptides for drug delivery to the wound bed from matrices, retargeted bacteriophages themselves may have intrinsic utility. There is a growing interest in the use of phage in the wound bed to control infection and biofilm development [62,63]. Indeed, Markoishvili *et al *[15] developed a novel sustained-release matrix using biodegradable polyester amides that are impregnated with bacteriophage and antibiotics to show efficacy in the management of infected venous stasis ulcers and other poorly healing wounds. Whether the peptides identified by phage display could enhance these sustained-release pharmacokinetics remains to be established. If so, they would require minimal reformulation. There are also significant advantages to the concept of using phage display peptides in the same structural context in which they were originally identified (that is, on the particle). It is for these very reasons that phage-based vectors for gene delivery have been put forth for development [64-66].

As emphasized by the changes we describe in the temporal and spatial distribution of VEGFR2 and SBE promoter activity after grafting (Figure 2), it is particularly apparent that one of the major hurdles faced by the field of tissue repair and regeneration is pharmacokinetic: different agents need to be delivered to different places at different times, depending on the state of repair [6,9,12]. This is a formidable challenge to regenerative sciences, because different agents also have different functions, depending on the context in which they are recognized by the cell. It is in view of this intrinsic complexity that deployment of a combinatorial method such as phage display to a combinatorial problem such as wound healing presents a novel approach to the clinical problem of tissue repair. It is interesting to speculate that emerging technologies such as *in vivo *biopanning may help exploit the intrinsic complexity of the wound-healing response for drug deployment. The studies presented here present a combinatorial approach towards targeting that uses traditional biopanning and peptide-selection techniques of phage display. Whether or not these techniques or other combinatorial methods (for example, using directed evolution) might enable the design and creation of the next-generation matrices remains to be determined. In the meantime, it will be important to evaluate the potential of matrix-targeting peptide candidates such as those described here for their utility in spatiotemporal drug delivery to the wound bed.

## Conclusions

We demonstrate how phage-display technologies can be used to identify peptide sequences that interact with Integra^® ^grafting matrices. We show that when multiple strategies are deployed in different classes of screening assays, they identify different classes of targeting peptides, which may each have differential value in the temporal-spatial targeting of drugs, biotherapeutics and nanoparticles to the wound bed.

## Methods

### Characterization of gene expression after full-thickness injury

#### Mouse graft model

All procedures were approved by the Institutional Animal Care and Use Committee at University of California, San Diego. Mice were anesthetized with isoflourane, then the dorsum of the animal was shaved to clear the field of fur and the surgical site prepared using aseptic techniques. A full-thickness circular wound measuring 15 mm in diameter was marked to the right of midline using a standard template, and the skin, subcutaneous tissue and fascia were excised. Integra^® ^grafts of 15 mm in diameter were cut from aseptic sheets and secured in place with approximately seven 3-0 silk sutures. Immediately after surgery, animals received 1.4 ml normal saline resuscitation and 0.05 mg/kg buprenorphine subcutaneously. Animals were allowed to recover under observation, returned to the vivarium, checked daily and maintained in a 12-hour light/dark cycle with free access to food and water.

#### Transgenic mouse models of gene expression

Male transgenic mice expressing firefly luciferase under the regulation of SBE (Jackson Laboratories, Bar Harbor, ME, USA) and transgenic mice expressing firefly luciferase under the regulation of the VEGFR2 promoter (kind gift of Dr. J. Molkentin; PMID 14656927) were used. The mice ranged in age from eight to 16 weeks old, and were handled as described previously [21].

#### Non-invasive in vivo imaging of gene expression during wound healing

Mice were anesthetized with isofluorane and given intraperitoneal injection with 1.5 mg of the substrate D-luciferin (Caliper Life Sciences, Hopkinton, MA, USA) in 150 μl saline. After a 5-minute incubation to obtain steady-state kinetics of substrate distribution, the still-anesthetized mice were imaged (Lumina CCD Imaging System' Caliper Life Sciences) according to the manufacturer's recommendations. Exposure-matched images were acquired (Living Image software, version 3.0; Caliper Life Sciences). All animal images shown in this study were exposure-matched (that is, matching color bar upper and lower limits in each panel). Regions of interest (ROI) showing bioluminescence in the wound area and matched sizes in the flank were used for image acquisition [21]. Each image was quantified in units of photons/sec/cm^2^/steradian to obtain a fold change as defined by wound divided by control ROI signal. Images were collected on days 3, 7 and 14 after grafting.

### Phage display libraries

As specifically indicated in Table 1, one of two commercial peptide libraries (New England Biolabs, Ipswich, MD, USA) were used. These libraries consisted of either 1) a disulfide-constrained heptapeptide (PHD-C7C) or 2) a dodecapeptide (PHD12) library. The randomized peptide segment of the PHD-C7C library is flanked by a pair of cysteine residues, which results in the display having peptide loops rather than linear sequences. All of the libraries have reported complexities in excess of 2 × 10^9 ^independent clones, and are displayed at the N-terminus of the minor coat protein pIII on M13 phage, at a valency of five peptides per virion. Unlike the PHD12 libraries, the first randomized position in the PHD-C7C library is preceded by Ala-Cys, and all of the libraries contain a short linker sequence (Gly-Gly-Gly-Ser) between the displayed peptide and pIII.

### Biopanning strategies for matrix binding peptide identification

#### Screening protocol 1: Intact matrix biopanning using different elution strategies

The first protocols (IT1) (Table 1; Figure 1) involved screening the PHD-C7C library on intact Integra^® ^bilayer matrix wound dressings (catalog number BMW4101, lot number 465012B; Integra^®^) that were first cut to fit into 24-well cell culture plates. Non-specific binding to these membranes was blocked by an overnight incubation with 5 mg/ml of BSA in PBS-T at 4°C. The following day, the blocking buffer was aspirated and washed in PBS-T. As indicated, the matrices were then incubated for 1 hour at room temperature with 2 × 10^12 ^wild type M13 phage [66,67] in 1% BSA to further block non-specific phage particle binding and enhance biopanning for displayed peptides. After washing these blocked matrices three times with PBS-T, 2 × 10^11 ^particles from the PHD-C7C library were added for 1 hour at room temperature in 1% BSA. Matrices were then processed with four washes of 1) PBS-T, 2) salt (0.5 mol/l NaCl) or 3) 0.2 mol/l glycine-PBS (pH 2.2) in 1% BSA. Particles remaining in the matrix were homogenized, and the recovered phage from each eluate was amplified, titered and further processed for the next round of screening. This process was repeated four times, after which the peptides were sequenced. Recoveries increased 40-fold over four rounds of biopanning.

#### Screening protocol 2: dissociated matrix biopanning

IT6 and IT7 were adapted from the protocol described above but used the PHD-C7C and PHD12 libraries, respectively, on sonicated matrix homogenates instead of intact matrix. This biopanning was specifically designed to address the high non-specific background seen with protocol 1, presumably a result of sequestration in the fenestrations of the intact matrix. First, 24-well plates were coated with 100 ul of dissociated Integra^®^, which was prepared by homogenizing a 30 × 40 mm matrix in 50 ml PBS at 4°C, and adding 100 μl of a 1:5 diluent to coat wells overnight at °C. The following day, the wells were washed with PBS-T and blocked by the addition of 0.1% BSA in PBS-T for 1 hour at room temperature. After washing four times with PBS-T, phage was added for 1 hour at room temperature. Unbound particles were removed with further washes, and binding assessed with and without 0.2 mol/l glycine (pH 2.2) acid wash to evaluate binding affinity. Phage that acquired binding capacity were collected at the first round using 1) chondroitin sulfate 100 μg/mL in PBS, 2) 0.2 mol/l glycine in 1% BSA or 3) 0.2 mol/l glycine/0.5 mol/l NaCl washes, or from the particles bound to matrix after all washes. Each collection was then cycled through three or four more rounds of selection using the appropriate elution strategy (Table 1), and peptide sequences obtained to monitor library collapse to binding sequences.

#### Screening protocol 3: cryostat section biopanning

The third protocol deployed (IT8) was developed to identify binding peptides capable of binding directly to matrices, avoiding bias for the selection of peptide sequestration while retaining *in situ *biopanning on matrix sections. To this end, matrices were frozen in optimal cutting temperature (OCT) compound, and 10 μm sections were prepared using a cryostat and then placed onto frozen slides. These sections were thawed, washed and incubated with the PHD-C7C library as described in protocol 1, and the bound phage eluted from sections with 1) chondroitin sulfate, 2) high salt (0.5 mol/l NaCl) or (3) 0.2 mol/l glycine (pH 2.2) in 1% BSA. The particles obtained in rounds 3 and 4 were used for sequencing.

#### Protocol 4: biopanning with and without NFM blocking

To address the possibility that selection bias was introduced by the nature of the blocking buffer, NFM was evaluated as a blocking agent in the IT9 screen to minimize non-specific binding. The profile of the targeting peptide candidates recovered was then compared with the use of PBS-T blocking buffer alone. In these experiments, the sonicated samples were processed into assay plates as above, but were either blocked during the pre-incubation with 1% non-fat milk in PBS-T, or with PBS-T alone. After 1 hour incubation with the PHD-C7C libraries, unbound peptides were removed with four PBS-T, washes and bound phage were eluted with either 0.2 mol/l glycine (pH 2.2) in 1% BSA or 0.2 mol/l glycine/0.5 mol/l NaCl to distinguish different classes of binding peptides that would have different affinities and different kinetics of release from intact grafts.

### DNA sequencing, peptide sequence deduction, homology and phylogeny analyses

DNA sequencing to deduce the peptide inserts encoded between the *Kpn*1/*Acc*65 1 and *Eag *1 restriction sites was performed as described by the manufacturer (New England Biolabs, Ipswich, MA, USA). Single-strand phage was prepared with a commercial kit (Spin M13 Kit; Qiagen, Valencia, CA, USA). After quantification of yields on agarose gels, DNA sequencing was performed using primers supplied by the manufacturer (CAACAAATCGTTTTAGGGTATGT) and (GCAATGCGATTGATACTCCCG). Data was analyzed using MacVector (Eastman Chemical Co., New Haven, CT, USA) and Vector NTI sequence analysis programs (Invitrogen, Carlsbad, CA, USA).

### ELISA and IT peptide validation

To characterize the binding specificity of candidate IT peptides, we developed modified ELISAs using both 96-well and 24-well microtiter plate formats. Plates were coated with 100 μl of dissociated Integra^® ^(prepared by homogenizing a 30 × 40 mm matrix in 50 ml PBS at 4°C and adding 100 μL of PBS-T diluent (1:5)) to coat wells overnight at 4°C. The following day, the wells were washed with PBS-T, and non-specific binding blocked by the addition of 0.1% BSA, non-fat milk or no protein in PBS-T for 1 hour at room temperature. After washing the wells four times with PBS-T, candidate phage were added for 1 hour at room temperature. Unbound particles were removed with further washes with PBS-T. Bound phage were detected by incubation for 2 hours with a horseradish peroxidase (HRP)-conjugated anti-M13 antibody (1:5000; MoBiTek, Gottingen, Germany), four washes with PBS-T and development of the HRP signal with chemiluminescence (Chemiglo; Alpha Innotech, San Leandro CA, USA).

To demonstrate spatially controlled binding of targeted particles, matrices were evaluated by imaging sections prepared in OCT. Negative controls (PBS) or targeted particles were deposited onto matrices in 5 μl PBS (10^13 ^particles/ml) as either a dot or a continuous line, and allowed to bind for 90 minutes at room temperature. After rinsing and blocking with 0.1%milk in PBS-T for 1 hour, the sections were incubated with anti-M13 HRP-conjugated antibody (1:5000) for 1 hour at room temperature. Signal was detected using chemiluminescence and visualized (IVIS Lumina Xenogen Imaging system; Caliper Biosciences).

### Statistical analysis

The change of bioluminescence after luciferin injection in wounds was analyzed in at least four animals in each group at each time point to obtain statistical significance. Student's *t*-test was used to analyze the data, and *P *< 0.05 was considered significant.

## Declaration of competing interests

The authors declare that they have no competing interests.

## Authors' contributions

RS performed the phage biopanning, screening and sequencing experiments, and contributed to the manuscript. CYP performed the validation experiments and contributed to the manuscript. AMG assisted in design selection, protocols for analyses and interpretation of data. BMM contributed to the conception, design and coordination of the study. BP coordinated sample acquisition and assisted with experimental design. RC contributed to the coordination and design of the study, and the editing of the manuscript. BE contributed to the conception, design and coordination of the study, and performed the *in vivo *experiments. AB conceived of the study, and contributed to the conception, design and coordination of the work, and the writing and revising of the manuscript. All authors read and approved the final manuscript.
